# Double burden: microfilariae infection amplifies metabolic costs of moult in breeding male village weavers (*Ploceuscucullatus*)

**DOI:** 10.1016/j.bbrep.2026.102576

**Published:** 2026-04-06

**Authors:** Felix A. Andong, Ezekiel S. Mayowa, Praise O. Nwanozie, Vincent C. Ejere, Abdifatah Ahmed A. Afyare

**Affiliations:** a Department of Zoology and Environmental Biology, Faculty of Biological Sciences, University of Nigeria, Nsukka, Enugu State, Nigeria; b AP Leventis Ornithological Research Institute, Faculty of Natural Sciences, University of Jos, Plateau State, Nigeria; c Department of Social Sciences , Faculty of Economics and Management Science, Salaam University, Mogadishu, Somalia

**Keywords:** Village weaver, Microfilariae infection, Energy metabolism, Ketone bodies, Triglycerides, Body condition index (BCI), Moult status, Life-history trade-offs

## Abstract

Breeding male birds face high energetic demands due to simultaneous investment in reproduction and feather moult, yet the metabolic consequences of parasitic infection during this period are poorly understood. To address this gap, we focused on non-moulting and actively moulting breeding adult male village weavers (*Ploceus cucullatus*) to investigate how microfilariae infection affects host biochemical energy status and overall condition. Using plasma glucose, triglycerides, β-hydroxybutyrate, and body mass adjusted for structural size as integrative markers, we examined how infection influences energy allocation and imposes physiological costs during this critical life-history stage. Specifically, we aimed to: (i) determine whether microfilariae infection and active moult influence short-term energy availability by examining plasma glucose concentrations, and whether absolute body mass modulates the effect of infection; and (ii) evaluate the combined and independent effects of infection and moult on lipid and ketone metabolism, while incorporating absolute body mass and size-corrected body condition index (BCI) to assess overall energetic reserves and physiological trade-offs. A total of 128 breeding males were trapped and screened for microfilariae and moult status. Our results indicate infected birds that are actively moulting experienced higher β-hydroxybutyrate, lower glucose and reduced BCI, when compared with the non-infected birds that were non-moulting. On the other hand, non-infected male birds that were also non-moulting maintained higher triglyceride levels. Our regression analyses indicate both infection and moult independently increased ketone concentrations and decreased triglycerides (P < 0.05), with no significant interaction for most markers. However, for β-hydroxybutyrate, the interaction may approach significance (P = 0.08), which suggest a marginal tendency toward non-additive effects. These results highlight a ‘double burden,’ where concurrent parasitism and moult constrain energy allocation, shifting metabolism from carbohydrates toward lipid catabolism. This study may provide mechanistic insight into how microfilariae infection amplifies energetic costs during high-demand life-history stages in breeding male village weavers.

## Highlights

• Breeding male village weavers with infection and active moult showed higher β-hydroxybutyrate and lower glucose and body condition.
• The effect of infection on glucose varied with body mass, with smaller infected males tending to have lower glucose than larger ones.
• Plasma triglycerides tended to be lower in birds experiencing infection or moult, suggesting increased reliance on lipid reserves.
• Most effects of infection and moult on metabolic markers appeared additive, with β-hydroxybutyrate showing only a weak tendency for interaction.
• Infection was treated as a binary variable, and variation in intensity was not measured, which is a limitation; robust regression helped reduce the influence of extreme values.

## Graphical abstract

**Figure:**
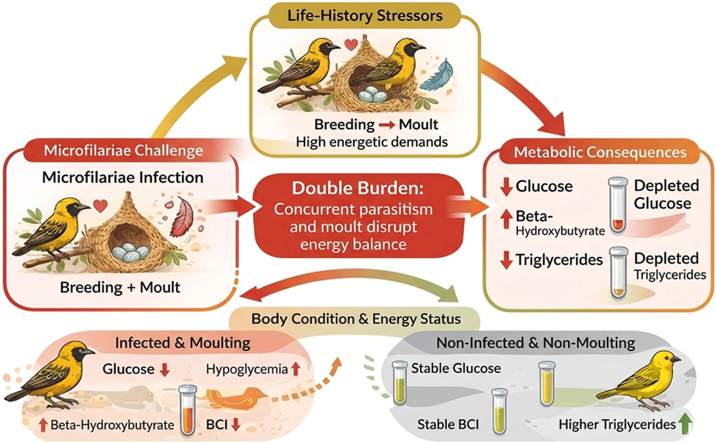

## 1 Introduction

In sexually dimorphic male birds, the breeding moult involves feather replacement that enhances visually conspicuous plumage signals used in mate attraction [1,2]. However, moulting during the breeding period is energetically costly, as feather synthesis requires substantial investments of amino acids, lipids and metabolic energy that occur concurrently with other reproductive demands [[3], [4], [5]]. Reproductive activities such as territory defense, nest building, mate guarding and courtship displays further increase activity levels and elevate basal metabolic rate in breeding males [6]. For these reasons, most breeding males carefully schedule moulting to minimize overlap with energetically demanding reproductive activities [5].

The combined energetic demands of breeding and moulting drive trade-offs among maintenance, growth and reproduction. Male birds that experience delayed moults or cannot efficiently balance these demands must strategically mobilize and allocate energy from internal reserves and dietary intake to sustain performance without compromising survival. Delays in moult have been reported in passerines infected with blood parasites [7,8]; however, it remains unclear whether microfilariae infections, generally considered nonlethal in birds, exacerbate energetic challenges during breeding.

In adult village weavers (*Ploceus cucullatus*), microfilariae are vector-borne nematode parasites residing in the bloodstream [9]. As with other blood parasites, microfilariae likely trigger immune activation, which carries energetic costs because leukocyte proliferation and acute-phase protein synthesis require ATP and nutrient substrates [10,11]. Thus, mounting an immune response may divert resources from reproduction and other physiological functions, imposing additional energetic constraints on infected hosts [12].

Parasitic infections in birds can alter body condition, affect immune function and raise circulating metabolites associated with energy mobilization [[13], [14], [15]]. These effects are often reflected in biochemical markers, such as plasma glucose, which indicates short-term energy availability and is tightly regulated to meet immediate metabolic demands [16,17]. During sustained energy demand, glucose can be supplemented by mobilized lipids measured as plasma triglycerides, which reflect both dietary fat intake and endogenous lipid stores [18]. When glucose is insufficient, ketone bodies such as β-hydroxybutyrate are produced from fatty acid oxidation to serve as alternative fuels [19]. In addition, body condition index (BCI), calculated from mass relative to structural size, provides an integrative measure of overall energetic reserves that captures cumulative effects of energy intake, expenditure and storage in muscle and fat [20].

Despite the relevance of metabolic reallocation during parasitic challenge, few studies have quantified the energetic consequences of microfilariae infection in wild passerines across life-history stages of the birds’ high energetic demand of moulting and breeding. This is because most avian parasitology research has focused on infection prevalence, immune response or reproductive output in isolation [13,21], leaving a gap in understanding how parasitic infections interact with host energy metabolism to shape physiological trade-offs.

To address this gap, we focused on nonmoulting and actively moulting breeding adult male village weavers to investigate how microfilariae infection affects host biochemical energy status and overall condition. Using plasma glucose, triglycerides, β-hydroxybutyrate and body mass adjusted for structural size as integrative markers, we examined how infection influences energy allocation and imposes physiological costs during this critical life-history stage.

Specifically, we aimed to: (i) Determine whether microfilariae infection and active moult influence short-term energy availability by examining plasma glucose concentrations, and whether absolute body mass modulates the effect of infection on circulating glucose levels; and.

(ii) Evaluate the combined and independent effects of microfilariae infection and moult on lipid and ketone metabolism by analyzing plasma triglycerides and β-hydroxybutyrate concentrations, while incorporating both absolute body mass and size-corrected BCI to assess overall energetic reserves and physiological trade-offs.

Because breeding, feather synthesis, and immune activation are energetically demanding processes that compete for finite resources, they create physiological trade-offs in energy allocation. Actively moulting males must invest in feather synthesis while sustaining reproductive activities under elevated energetic demand. If microfilariae infection increases metabolic expenditure via immune activation, infected individuals are expected to experience additional energetic disruption. We therefore hypothesized that microfilariae infection and active moult might independently and interactively influence metabolic indicators of energy allocation. Specifically, we predicted that infection and moult would alter plasma glucose concentrations, with absolute body mass modulating infection-related variation. We further predicted that infection and moult would drive shifts in lipid and ketone metabolism, reflected in increased β-hydroxybutyrate, altered triglycerides, and reduced energetic reserves. Individuals experiencing both stressors concurrently were expected to show the strongest metabolic shifts, consistent with additive energetic costs. By examining both absolute body mass and size-corrected BCI, we capture complementary insights into immediate energy availability and relative energetic reserves during these energetically demanding life-history stages. Nonetheless, these insights remain observational and do not demonstrate direct causality.

## 2 Materials and methods

### 2.1 Ethical approval

This study was approved by the Scientific Committee of the A.P. Leventis Ornithological Research Institute, University of Jos, Plateau State, Nigeria. All field procedures were conducted in accordance with the Institute's ethical and institutional guidelines for the use of wild birds in research.

### 2.2 Bird trapping

Fieldwork was carried out in 2023 during the peak breeding season of the village weaver, from June to August, at the Amurum Forest Reserve of the A.P. Leventis Ornithological Research Institute (09°89′ N, 08°97′ E; Plateau State, Nigeria). Adult male village weavers were captured using mist nets between 06:30 and 10:00 h. Upon capture, each bird was fitted with a uniquely numbered aluminium ring to allow individual identification and prevent resampling.

### 2.3 Assessment of actively moulting and non-moulting birds

Adult male village weavers exhibit marked sexual dichromatism. Prior to the breeding season, males ready to breed typically undergo a partial prenuptial moult of the head and body feather tracts, which is completed by the peak of the breeding period [9]. This moult results in pronounced changes in plumage, characterized by brighter yellow body feathers contrasted with a conspicuous black facial mask and throat [22,23].

For this study, our primary targets were breeding adult male village weavers fully into breeding plumage. We specifically focused on individuals that were either actively moulting or non-moulting, as our main interest was to examine the additive energetic burden associated with active feather replacement during breeding. Adult males displaying breeding plumage but still undergoing moult in one or more feather tracts were classified as actively moulting, whereas individuals showing no evidence of moult in any of the assessed body regions were classified as non-moulting. Within these two categories, we further distinguished between infected and non-infected birds to evaluate how parasitic infection interacts with moult to influence energetic status.

### 2.4 Assessment body condition

Similar to previous studies [24,25], body condition was assessed using a body condition index (BCI), calculated by adjusting body mass for structural size. Nonetheless, raw body mass was also examined to test whether absolute mass interacts with infection or moult to influence plasma glucose. This approach allowed comparison of size-corrected and absolute effects, to provide a complete understanding of energy allocation under parasitic and moulting stress. Body mass was measured to the nearest 0.1 g using a Meubon® digital electronic balance (Model: ACS 30 JE11). Wing length, used as an index of structural size, was measured to the nearest 0.1 mm with a metal ruler. BCI was then computed from these values, giving a size-corrected estimate of body condition that accounts for individual structural differences [25].

### 2.5 Blood collection

Blood was collected within 3 min of capture by pin-pricking the brachial vein with a sterilized lancet, and approximately 75 μL of blood was collected using a microhaematocrit capillary tube. Thin blood smears were then prepared for filarial screening [26].

### 2.6 Assessment of plasma glucose and β hydroxybutyrate

To prevent post collection degradation, plasma glucose was measured immediately in fresh samples using a CentriVet point of care device. Glucose strips were inserted into the instrument, and the strip tip was brought into direct contact with the sample. Plasma glucose concentration was determined by measuring the electrical current generated by the enzyme substrate reaction on the strip, with a measurable range of 10 to 600 mg dL^−1^ [27,28]. Beta hydroxybutyrate concentration was measured from whole blood by placing a single drop onto disposable test strips compatible with the CentriVet GK device. Measurements were taken after calibration of the device according to the manufacturer's instructions. The instrument provides beta hydroxybutyrate readings ranging from 0.0 to 8.0 mmol L^−1^ within 10 s [28].

### 2.7 Assessment of plasma triglycerides

Triglyceride concentrations were measured using a CardioChek device with reagent strips, which have a detection range of 50 to 500 mg dL^−1^. The device uses light reflectance technology to quantify an enzymatic reaction triggered when blood is applied to the test strip. This reaction produces a color change on the strip, and the analyzer measures the light reflected from the underside of the application window. The intensity of the color change is proportional to the triglyceride concentration in the sample. However, when blood samples showing incomplete color change, we discarded them to ensure measurement accuracy [29].

### 2.8 Screening for microfilariae

Microfilariae were screened using thin blood smears. Smears were air dried for 15 min, fixed in absolute methanol for 2 min, and then stained with Giemsa for 30 min. Each smear was examined under oil immersion at 1000 × magnification using a Nikon microscope. Microfilariae were identified based on morphological characteristics [30].

### 2.9 Data analyses

All statistical analyses were conducted in *R* software to examine the effects of microfilariae infection, moult status and energetic condition on short-term energy availability and metabolic biomarkers in breeding adult male village weavers. Plasma glucose, used as a proxy for short-term energy availability, was first tested for normality (Shapiro-Wilk test, *P* = 0.528) and found to be normally distributed. A linear regression model was then fitted with plasma glucose as the response variable and infection status, moult status, body mass and their interaction (infection × body mass) as predictors. Non-significant terms (*P* > 0.05) were sequentially removed using stepwise backward elimination, beginning with interaction terms.

To evaluate lipid and ketone metabolism, a robust regression models (*lmrob*, *robustbase* package) were fitted separately for plasma triglycerides, β-hydroxybutyrate and plasma glucose. Predictors included infection status, moult status, BCI and the infection × moult interaction. BCI was calculated as the residuals of body mass regressed on wing length to provide a size-corrected estimate of energetic reserves. Nonetheless, the model framework allowed us to identify significant main effects and interactions while accounting for energetic condition and physiological trade-offs across multiple metabolic pathways.

Although infection status was treated as a binary variable (infected vs. non-infected) due to sampling and detection constraints, data on the number of microfilariae per individual were not available. Robust regression was employed to account for outliers in metabolic markers, ensuring that extreme values did not unduly influence model estimates.

## 3 Results

In total, 128 breeding adult male village weavers were trapped, comprising actively moulting and infected (35 birds, 27%), actively moulting and non-infected (25 birds, 20%), non-moulting and infected (31 birds, 24%), and non-moulting and non-infected (37 birds, 29%). Microfilaria-infected males undergoing moult experienced the highest β-hydroxybutyrate levels (0.93 mmol L^−1^), but their plasma glucose concentrations (8.10 mg dL^−1^) and body condition index (BCI) values (−0.05) were the lowest. In contrast, non-infected, non-moulting individuals maintained the highest triglyceride concentrations (1.54 mmol L^−1^) and stable glucose levels (10.8 mg dL^−1^) (Table 1).

**Table 1:** Mean physiological marker concentrations and body condition index (BCI) in village weavers grouped by infection and moult status. This table highlights how the presence of microfilariae and the energy-intensive process of growing new feathers (moult) relate to the birds' energy reserves during breeding.

Group	(%) of total birds trapped	Glucose (mg dL^−1^)	Triglycerides (mmol L^−1^)	β-Hydroxybutyrate (mmol L^−1^)	BCI
Infected and Moulting	35 (27)	8.10	1.45	0.93	−0.05
Infected and Non-Moulting	31 (24)	8.90	1.41	0.84	−0.02
Non-Infected and Moulting	25 (20)	11.0	1.52	0.79	0.08
Non-Infected and Non-Moulting	37 (29)	10.8	1.54	0.85	0.04

**Fig. 1:**
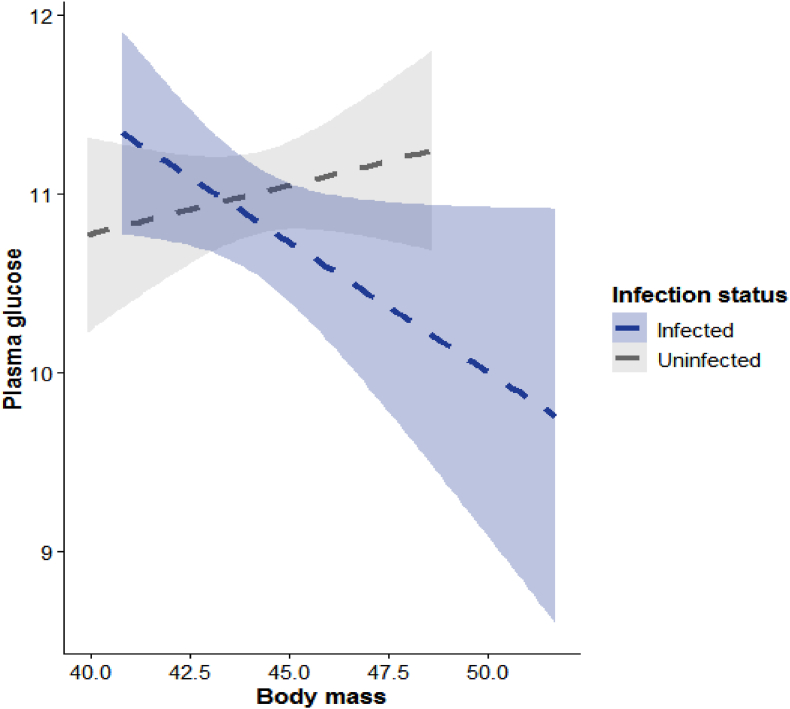
Relationship between microfilariae prevalence and body mass on plasma glucose levels. Indicating how body mass and plasma glucose concentrations is modified by infection status.

The linear model indicates infection status significantly increased plasma glucose (β = 8.65, *P* = 0.04). Body mass was also a significant positive predictor of plasma glucose (β = 0.25, *P* = 0.02), whereas moult status had no significant effect. However, there was a significant interaction between infection × body mass (β = −0.20, *P* = 0.03), indicating that the positive effect of infection on glucose is stronger in birds with lower body mass and diminishes as body mass increases (Table 2 and Fig. 1).

**Table 2:** Parameter estimates from the final linear regression model examining the effects of infection status, moult status, body mass, and their interaction on plasma glucose concentrations in breeding adult male village weavers. Significant predictors are shown in bold.

Variable	β	SE	*t*	P
**Plasma glucose**				
Intercept	**8.60**	**2.68**	**3.21**	**0.01**
Infection	**8.65**	**4.07**	**2.13**	**0.04**
Moult (yes)	−0.01	0.20	−0.04	0.97
Body mass	**0.25**	**0.08**	**3.13**	**0.02**
*Infection × Body mass*	**−0.20**	**0.09**	**−2.16**	**0.03**

**Fig. 2:**
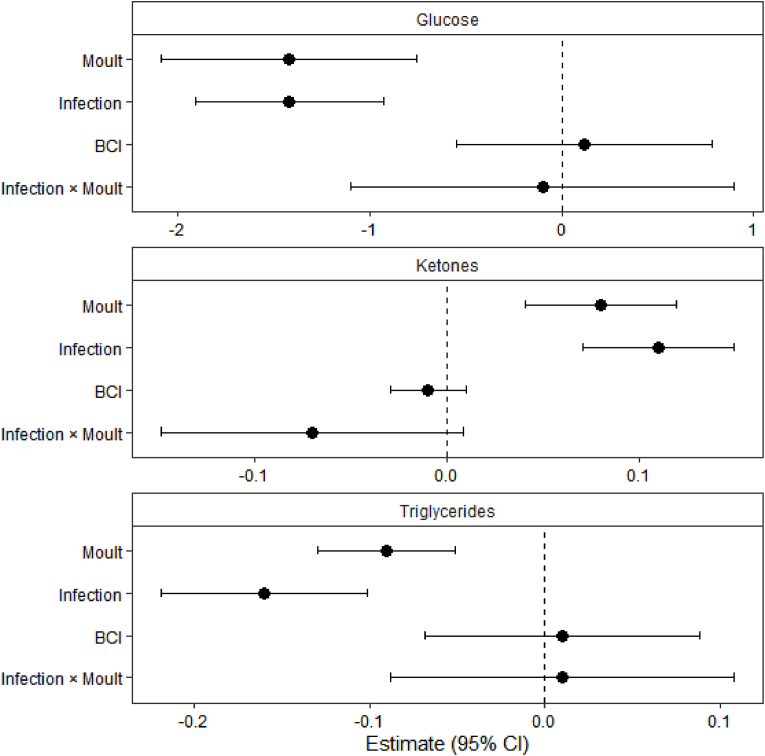
Physiological and metabolic impacts of moult and microfilariae on energetic reserves and body condition. This figure illustrates the effects of moult status, filarial infection, their interaction, and Body Condition Index (BCI) on key physiological markers: plasma glucose, β-hydroxybutyrate (ketones), and triglycerides. Black dots represent estimated effect sizes, indicating the direction and magnitude of each predictor, and horizontal lines show 95% confidence intervals. The vertical dashed line at zero indicates no effect; confidence intervals crossing this line suggest non-significant effects, whereas estimates entirely to the left or right indicate significant negative or positive effects, respectively.

On the other hand, the robust regression model indicates infection status significantly decreased plasma triglycerides (β = −0.16, *P* < 0.001). Moulting status also significantly reduced triglyceride concentrations (β = −0.09, *P* < 0.001), whereas body condition index (BCI) had no significant effect (*P* = 0.80). There was no significant interaction between infection × moulting (β = 0.01, *P* = 0.84), indicating that the effect of infection on triglycerides did not differ between moulting and non-moulting birds (Table 3a and Fig. 2).

**Table 3:** Parameter estimates from robust regression models examining the effects of infection status, moult status, body condition index (BCI) and their interaction on plasma triglycerides, β-hydroxybutyrate, and plasma glucose concentrations. Significant effects are highlighted in bold.

Variables	β	SE	*t*	P
**a) Triglycerides**				
Intercept	**1.58**	**0.02**	**79.0**	**< 0.001**
Infection	**−0.16**	**0.03**	**−5.33**	**< 0.001**
Moulting (yes)	**−0.09**	**0.02**	**−4.50**	**< 0.001**
BCI	0.01	0.04	0.25	0.80
*Infection × moulting (yes)*	0.01	0.05	0.20	0.84
**b) Ketones (β-hydroxybutyrate)**				
Intercept	**0.76**	**0.02**	**38.0**	**< 0.001**
Infection	**0.11**	**0.02**	**5.50**	**< 0.001**
Moulting (yes)	**0.08**	**0.02**	**4.00**	**< 0.001**
BCI	−0.01	0.01	−1.00	0.32
*Infection × moulting (yes)*	−0.07	0.04	−1.75	0.08
**c) Plasma glucose**				
Intercept	**11.1**	**0.17**	**65.3**	**< 0.001**
Infection	**−1.42**	**0.25**	**−5.68**	**< 0.001**
Moulting (yes)	**−1.42**	**0.34**	**−4.18**	**< 0.001**
BCI	0.12	0.34	0.35	0.72
*Infection × moulting (yes)*	−0.10	0.51	−0.20	0.85

For ketones, infection status significantly increased β-hydroxybutyrate concentrations (β = 0.11, *P* < 0.001), and moulting birds also experienced significantly higher ketone levels (β = 0.08, *P* < 0.001). BCI had no significant effect (*P* = 0.32). While the infection × moulting interaction was not statistically significant, the term may reflect a weak tendency toward non-additive effects (β = −0.07, *P* = 0.08). This distinction clarifies that most interactions were non-significant, and only β-hydroxybutyrate showed a marginal trend (Table 3b and Fig. 2).

Lastly, infection status significantly decreased plasma glucose concentrations (β = −1.42, *P* < 0.001). Moulting status was also associated with a significant reduction in plasma glucose (β = −1.42, *P* < 0.001), whereas BCI had no significant effect (*P* = 0.72). The infection × moulting interaction was not significant (β = −0.10, *P* = 0.85), indicating that the effects of infection and moult on glucose were independent (Table 3c and Fig. 2).

## 4 Discussion

In this study, while our findings are consistent with predictions regarding alterations in energy allocation, these associations should be interpreted with caution as they do not establish direct causality. Secondly, that our data demonstrate a clear association between microfilarial infection and reduced body condition, the observational nature of this research means we cannot definitively rule out the influence of confounding ecological factors. For instance, variables such as localized food scarcity, subclinical coinfections or varying levels of predation pressure may have independently contributed to the lower body mass observed in infected individuals [31]. Nonetheless, breeding adult male village weavers that were concurrently infected with microfilariae and undergoing moult experienced the highest β-hydroxybutyrate concentrations that was accompanied by reduced plasma glucose levels and lower body condition. This simultaneous occurrence likely represents a potential “double burden,” whereby glucose reserves may be insufficient to meet energetic demands, hence forcing birds appear to rely on endogenous fat stores to support both immune responses and feather growth.

In contrast, non-infected and non-moulting individuals maintained higher triglyceride concentrations and stable glucose levels. As such, these patterns suggest the combined energetic demands of infection and moult might impose a substantial metabolic burden specifically on males that are both infected and moulting **(**Table 1). In this study, the observed depletion of circulating lipids suggests that birds are consuming energy substrates faster than they can replenish them. This was supported by the significant negative effects of both infection and moult on triglyceride concentrations (Table 3a). As such, our trend suggest the confidence intervals for both filarial infected and moulting indivdiduals lie entirely to the left of the zero line, hence indicating a consistent reduction in lipid availability (Fig. 2). Such metabolic changes are similar to those observed in birds during other high-energy periods, such as migration or reproduction. During these times, birds must quickly break down and use their fat stores to power their increased physical activity [18].

Consistent with this interpretation, microfilariae are known to associate with higher glucose levels; however, in this study, the magnitude of this effect depends on the bird's body mass. These associations are correlational and do not establish direct causality, as indicated by the significant interaction term (Table 2 and Fig. 1). Hence, it is likely that the energetic consequences of infection on glucose availability are modulated by body condition, where larger infected males show a distinct glucose response compared with smaller individuals. This highlights that the metabolic effects of microfilarial infection are not uniform across individuals but interact with body reserves to influence energy allocation in male village weavers. Comparable shifts in plasma metabolites have been reported in wild passerines during energetically demanding phases such as moult, where declines in glucose and triglycerides reflect increased energy expenditure and increased reliance on lipid metabolism [32].

Our results further demonstrate that although higher body mass is typically associated with good health in birds [9], heavier filarial-infected male village weavers were nevertheless unable to maintain glycaemic balance (Table 3c and Fig. 2). Filarial infection appears to drive these individuals into a metabolic state characterized by rapid depletion of glucose reserves and a compensatory shift toward lipid catabolism (Table 3b and Fig. 2). In passerines and other small birds, increase levels of β-hydroxybutyrate reflects increased lipid catabolism from adipose tissue during periods of energetic stress, often indicating poorer condition or reliance on stored fuel [33]. However, the negative interaction between infection and moult for β-hydroxybutyrate suggests a physiological ceiling where further lipid mobilization is limited. The marked decline in plasma triglycerides further supports this interpretation; as circulating levels drop significantly under the ‘double burden’ of infection and moult, the bird may eventually face finite limits in its capacity to sustain elevated fat oxidation (Table 3a and Fig. 2).

Mostly, infection is known to induce profound alterations in whole-body metabolism, which is characterized by accelerated glucose and lipid fluxes [34]. In line with this framework, our models suggest a pronounced disruption of carbohydrate homeostasis, with both moult and filarial infection exerting strong negative effects on plasma glucose concentrations (Table 3c and Fig. 2). This decline suggests that the energetic demands associated with feather synthesis and immune activation exceed the birds’ capacity to maintain glucose availability. But then, the absence of a significant relationship between BCI and glucose (Table 3c) implies that hypoglycaemia arises as a direct physiological response to infection and moult, even in individuals with substantial energetic reserves, hence underscoring the intensity of the metabolic demands imposed by these stressors.

While the current study focuses on these metabolic shifts in glucose, triglycerides and ketone bodies, it is important to recognize that the physiological costs likely extend to other systems that might include oxidative homeostasis. The metabolic demands of mounting an immune response and synthesizing new feathers can lead to the overproduction of reactive oxygen species, potentially resulting in oxidative damage if not balanced by antioxidant defenses. As such, although the oxidative stress profiles of these birds are outside the scope of the present manuscript, incorporating such markers in future syntheses will be essential to fully understand the trade-offs between self-maintenance and immune function in an ecological context. This is particularly relevant given that oxidative stress can have cumulative, long-term impacts on passerines [35], and both the production of reactive oxygen species (ROS) and a host's capacity to manage them vary with developmental stage and environmental conditions [36]. Furthermore, even when certain birds invest heavily in maintaining oxidative balance, they may still possess relatively ineffective antioxidant defense systems [37]. Similar physiological costs have been observed in other taxa. For example, short-term fasting in amphibian larvae triggers significant oxidative stress and lipid damage due to an inadequate antioxidant response, a cost that is particularly severe in hybrid individuals and potentially reduces their survival in fluctuating environments [38].

## 5 Conclusion

Our study demonstrates that the combined energetic demands of breeding moult and microfilariae infection could impose substantial metabolic constraints on adult male breeding village weavers. Consistent with our hypotheses, males experiencing both stressors exhibited the most pronounced shifts in energy allocation that was characterized by hypoglycemia, elevated β-hydroxybutyrate and reduced triglyceride levels, hence reflecting a compensatory reliance on lipid catabolism when glucose reserves become insufficient. Also, absolute body condition had limited influence that may suggest observed metabolic shifts are primarily driven by the physiological costs of feather synthesis and immune activation rather than variation in energy reserves alone. While the interaction between moult and infection was non-additive for most markers, the pattern highlights a physiological ceiling in lipid mobilization under concurrent stressors, which is likely a “double burden” whereby simultaneous parasitism and moult constrain energetic flexibility. By quantifying plasma glucose, triglycerides, β-hydroxybutyrate and body condition index during a high-demand life-history stage, our study provides evidence for a potential mechanistic understanding of how parasitic infection can exacerbate energetic trade-offs in breeding male village weavers. Nonetheless, our findings emphasize that in sexually dimorphic male birds, the timing of energetically costly processes like moult and reproduction must be carefully balanced, and parasitic infections parasitic infections may be associated with increased physiological stress. Future research should expand to include females, juveniles, and additional metabolic and immune markers, as well as experimental manipulations and longitudinal monitoring, to fully capture the ecological and physiological consequences of concurrent parasitism and life-history demands. Moreover, while the current study focuses on metabolic markers, it is essential to explore oxidative stress profiles in relation to filarial infections in these passerines; integrating these datasets in future work will provide a more comprehensive view of the physiological trade-offs associated with concurrent moult and parasitism. Also, we note that treating infection as binary may oversimplify the energetic consequences of varying infection intensities. Birds with low versus high microfilarial loads may experience different metabolic costs, and future work should incorporate infection intensity to better quantify these effects.

## Consent to publish

All authors and participants granted permission for this research work to be published.

## Author contributions

FAA and VCE conceived and designed the study. FAA and ESM collected field data. POA, AAAA, FAA and ESM performed the laboratory analyses. FAA performed statistical design, data analyses and interpretation. VCE, POA, AAAA and FAA wrote manuscript. VCE, ESM and FAA revised the manuscript. All authors read and approved the final manuscript.

## Ethical approval

This study received approval from the scientific committee of the A.P. Leventis Ornithological Research Institute at the University of Jos, and the fieldwork adhered to the institutional guidelines.

## Ethical responsibilities of authors

All authors have read, understood, and have complied as applicable.

## Disclosure statement

The authors have declared that no competing interests exist.

## Funding

The current study was solely funded by the authors.

## Declaration of competing interest

The authors declare that they have no known competing financial interests or personal relationships that could have appeared to influence the work reported in this paper.

## Data Availability

All data will be available from the corresponding author upon reasonable request.
